# A simulation-based pilot study of crisis checklists in the emergency department

**DOI:** 10.1007/s11739-021-02670-7

**Published:** 2021-03-09

**Authors:** Beatrice Billur Knoche, Caroline Busche, Marlon Grodd, Hans-Jörg Busch, Soeren Sten Lienkamp

**Affiliations:** 1 Department of Emergency Medicine, University Medical Center Freiburg, Faculty of Medicine, University of Freiburg, Freiburg, Germany; 2 Present Address: Department of Gynaecology and Obstetrics, Vivantes Klinikum Am Urban, Berlin, Germany; 3 Department of Internal Medicine, Renal Division, University Medical Center Freiburg, Faculty of Medicine, University of Freiburg, Freiburg, Germany; 4 Institute of Medical Biometry and Statistics, Faculty of Medicine and Medical Center-University of Freiburg, Freiburg, Germany; 5 Institute of Anatomy, Faculty of Medicine, University of Zurich, Zurich, Switzerland

**Keywords:** Emergency medicine, Checklist, Simulation, Resuscitation

## Abstract

Checklists can improve adherence to standardized procedures and minimize human error. We aimed to test if implementation of a checklist was feasible and effective in enhancing patient care in an emergency department handling internal medicine cases. We developed four critical event checklists and confronted volunteer teams with a series of four simulated emergency scenarios. In two scenarios, the teams were provided access to the crisis checklists in a randomized cross-over design. Simulated patient outcome plus statement of the underlying diagnosis defined the primary endpoint and adherence to key processes such as time to commence CPR represented the secondary endpoints. A questionnaire was used to capture participants’ perception of clinical relevance and manageability of the checklists. Six teams of four volunteers completed a total of 24 crisis sequences. The primary endpoint was reached in 8 out of 12 sequences with and in 2 out of 12 sequences without a checklist (Odds ratio, 10; CI 1.11, 123.43; *p* = 0.03607, Fisher’s exact test). Adherence to critical steps was significantly higher in all scenarios for which a checklist was available (performance score of 56.3% without checklist, 81.9% with checklist, *p* = 0.00284, linear regression model). All participants rated the checklist as useful and 22 of 24 participants would use the checklist in real life. Checklist use had no influence on CPR quality. The use of context-specific checklists showed a statistically significant influence on team performance and simulated patient outcome and contributed to adherence to standard clinical practices in emergency situations.

**Supplementary Information:**

The online version contains supplementary material available at 10.1007/s11739-021-02670-7.

## Introduction

Checklists were first introduced in aviation decades ago as a cognitive aid to minimize human error during standardized procedures and since then, ever more industries, especially high reliability organizations, have benefited from the experience of checklist research [1, 2]. Recently checklists have also become the focus of medical studies [3, 4]. Placed in the right context, checklists have a huge potential for increasing patient security by reducing exposure to adverse events during hospitalization [5–8].

The most prominent example for checklist implementation in clinical use is the WHO Surgical Safety Checklist, which has shown to reduce postoperative complications and mortality significantly [9]. Since this publication in 2009, the number of studies describing checklist development in various medical fields has steadily increased [10–16].

Besides checklists for daily routines, there is strong interest in developing emergency checklists for critical and high hazard situations given the prevalent exposure to human error [17–20]. Even trained personnel are susceptible to cognitive impairment caused by stress during medical emergency situations resulting in higher rates of patient exposure to adverse events [21–23]. When set in the right context, professionally developed, and carefully implemented, checklists can be an effective method to countering these effects [3, 4, 24]. Hence, in the past decade, there have been several publications of pilot trialling checklists for different kinds of medical crisis situations [24–30].

Yet, emergency checklists for internal medicine are scarce, possibly because emergencies occur in diverse kinds of settings in comparison to, e.g., an operating theater with fixed procedures [12, 14] or the typical sequence of endotracheal intubation [25].

Therefore, we aimed to develop four crisis checklists for use in an emergency department focused on handling internal medicine cases and to evaluate the checklists’ effect on crisis intervention during four simulation scenarios with structured observation.

Our hypothesis is that our crisis checklists improve adherence to best practices and evidence-based medicine in high hazardous emergency situations, hence reduce adverse events and, in consequence, lead to an improved patient outcome in acute care medicine.

## Materials and methods

### Study design

We defined the following topics for our checklists: Cardiac arrest with cardiopulmonary resuscitation and approach to hemodynamic unstable cardiac arrythmias. Content was determined by extensive literature research to display up-to-date and evidence-based methods and procedures supported by careful review of our medical experts.

An open-source template by the American think tank *AriadneLabs* (Boston, Massachusetts, USA) was used for fundamental design [31] and was edited using the graphics program *Adobe Illustrator CS5®* (Adobe Systems Incorporated, San José, California, USA). Subsequently, we designed four checklists in German with adaption to local conditions following expert methodology [1, 32, 33]. Two checklists comprised the topic Cardiac arrest displaying the approach to shockable and non-shockable rhythms, respectively. A third checklist covered the topic Haemodynamic unstable Tachyarrhythmia, a fourth the topic Hemodynamic unstable Bradyarrhythmia. The checklists as used in the trial are provided in the Supplementary Material with an English translation.

Corresponding to the checklists, we developed four crisis scenarios and protocols which are also available in the Supplementary Material.

Teams of four participants spent a session of three hours in a simulated emergency room with a simulator manikin (ResuscieAnne® QCPR, Laerdal Medical GmbH Deutschland, Puchheim, Germany) being confronted with these crisis scenarios. In half the scenarios, the teams had access to a binder format booklet with the four checklists. All teams were confronted with the same scenarios and had access to the same checklists. The order of the scenarios was arranged randomly and in which scenario a checklist could be used was determined by the study committee beforehand. No indication as to which of the 4 available checklists was relevant to the current scenario was given.

Participants entered as single individuals and were allocated to their teams by fixed dates determined by the study committee. Characteristics did not account for team allocation. Each team appointed a leader and a checklist reader prior to starting the session. The team leader stayed in his or her role throughout the session; whereas, the checklist reader became a normal participant in the scenarios without a checklist.

Every scenario was preceded by a patient history. After this, teams were informed if a checklist was available in this scenario. They had 8 min per scenario to reach the primary endpoint. Access to diagnostics and the patient history was granted and vital parameters were constantly displayed on a patient monitor.

A questionnaire with 43 topics presented in a 4-point Likert scale was handed to each participant after each scenario to collect their experience regarding the checklists and the scenarios. Each scenario was video-recorded for later review (Camcorder HDR-CX240E, Sony Corporation, Tokyo, Japan).

The study was carried out over a period of 24 months.

### Study participants

All study participants were medical students at a German university and had to have a minimum medical education of 2 years which includes training in advanced life support courses. Field training in emergency medicine was not a necessary requirement. Participation was voluntary, took place outside of the regular medical curriculum and was not compensated. Each study participant attended only one study session and gave prior written informed consent including consent to publish. Participants had no prior access to or knowledge of the checklists, the content of the study, the crisis scenarios, or the scenario order. An example checklist was presented in an introductory presentation to familiarize the participants with the general structure of the checklists.

### Data acquisition

Data were collected and analyzed using Microsoft Excel® (Microsoft Corporation, Redmond, Washington, USA). “Primary endpoint” was defined as the combination of establishing hemodynamic stability and declaration of the underlying diagnosis by one of the team members. The scenario was ended as soon as the primary endpoint was reached or after 8 min by us.

We determined secondary endpoints for each scenario which represent key process steps that significantly influence patient outcome according to current literature. 108 key processes were defined by the study committee, their merit displayed by a point system in which every key process holds a point value corresponding to its individual significance, resulting in a total score of 704 points to be acquired by each team in one session (a list of all key processes per scenario, their assigned point values and corresponding literature are provided in the Supplementary Material). Points were assigned during review of video recordings.

### Statistical analysis

For the primary endpoint, the odds ratio was calculated based on contingency tables (Supplementary Material) and Fisher’s exact test. To account for the fact that the observations are not independent since each team passed every scenario, we performed additional sensitivity analysis (Supplementary Material), i.e., a model fitted by generalized estimation equations (gee) with a logistic model to determine the effect of the checklist on the primary outcome. This approach is suitable to account for dependencies between observations by weighting these equations with a Covariance Matrix. This model is used for sensitivity reasons due to the small sample size. The secondary endpoint was tested by a multivariable linear regression, which was adjusted by Scenarios. This analysis was justified by additional sensitivity analysis: accounting for interactive effects between the scenario and the checklist and a random-effect model to quantify a possible effect of the team itself on the outcome (Supplementary Appendix). The odds ratio for the primary endpoint and regression parameters for the secondary endpoint were calculated each with a 95% confidence interval. Analyses and figures were generated using R (Version 3.6.1 (2019-07-05)).

## Results

### Participants’ characteristics

Six teams with 24 participants (four participants per team) completed 24 crisis sequences, 12 sequences with and 12 sequences without a checklist. Participants’ characteristics are displayed in Table 1. All participants were medical students in their 3rd year or above and presented a wide range of medical experience levels and knowledge regarding medical crisis situations. 42% of the participants had worked as paramedics for a year or more prior to or while studying; whereas, 46% had never been confronted with a real-life CPR situation before.

**Table 1:** Participants’ characteristics

	Answer option	No. (%) (*N* = 24)
Position	Student	24 (100%)
	Paramedics	10 (42%)
Year of Medical Study	3rd year	5 (21%)
	4th year	10 (42%)
	5th year	3 (12%)
	6th year	6 (25%)
Years of Experience in Emergency Medicine	0	14 (58%)
	1–3 years	4 (27%)
	4–6 years	4 (17%)
	> 7 years	2 (8%)
How often have you participated in a CPR-situation?	0	11 (46%)
	1 − 3*x*	3 (12%)
	4 − 6*x*	7 (29%)
	> 7*x*	3 (12%)
How often have you participated in an unstable arrhythmia situation?	0*x*	11 (46%)
	1 − 3*x*	11 (46%)
	> 4*x*	2 (8%)

### Primary and secondary endpoints

The combined primary endpoint of this study was reached in 8 of the 12 sequences with a checklist and in 2 of the non-checklist sequences resulting in an odds ratio of 10 (95% CI 1.11, 123.43; *p* = 0.03607, Fisher’s exact test) for reaching the primary endpoint when using the checklist in our crisis scenarios.

**Fig. 1:**
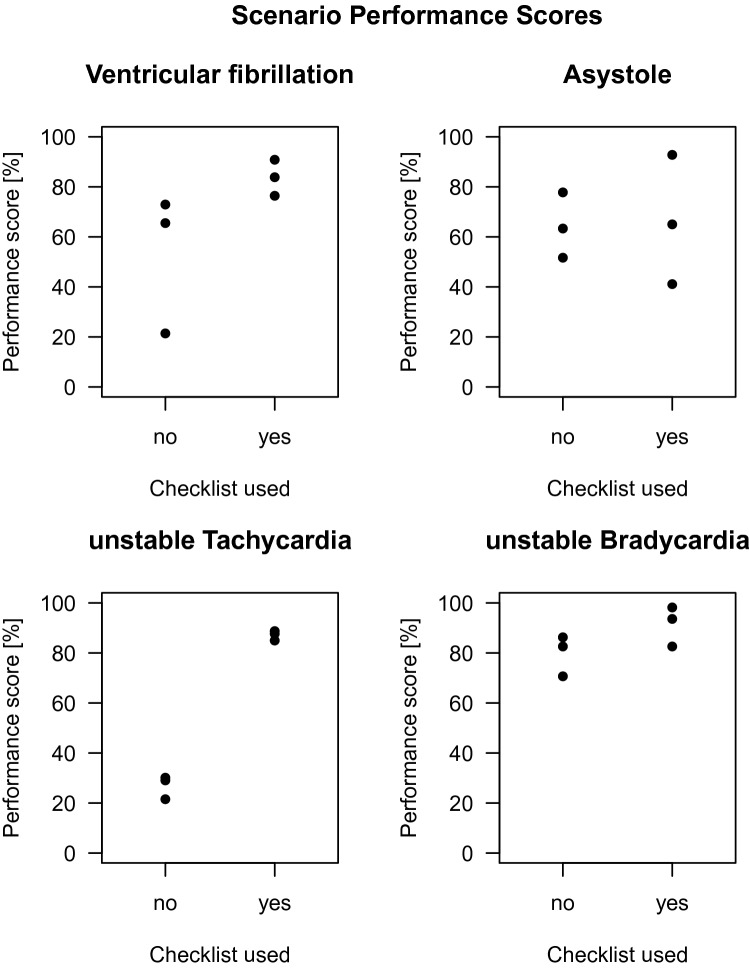
Performance score values according to scenario with and without checklist use. Each scenario was performed by six teams, three of each with or without access to the checklist

Adding together the point values of all key processes, a total of 704 points could be reached by each team throughout the session. In the 12 sequences with a checklist, participants reached an average of 573 (± 42) points or a performance score of 81.9% vs. an average of 376 (± 40) points or a performance score of 56.3% in the 12 sequences without a checklist (Fig. 1). Linear regression analysis identified a highly significant increase of 26% in the average performance score that could be attributed to use of the checklist (*p* = 0.00284).

In all four scenarios, more critical steps were taken, and higher point values reached using a checklist. However, the limited number of simulations tested does not allow for individual statistical analysis (Table 2). We note, that the most prominent effect was observed in the Haemodynamic unstable Tachyarrhythmia scenario. With the checklist, 22 of 27 (± 1) critical steps were taken and 162 of 186 point values reached, while only 8.3 (± 1.5) critical steps taken and 50 of 186 point values reached without a checklist present (performance score of 87.1% with checklist, vs. 26.9% without checklist) (Table 2).

**Table 2:** Average critical steps and point values given in total numbers reached with standard deviation listed by scenario and if checklist was used given in percentage of performance score

Scenario (maximum critical steps / point value)	Average critical steps taken / point value reached without checklist [± SD]	Average critical steps taken / point value reached with checklist [± SD]	Performance score without checklist (%)	Performance score with checklist (%)
Cardiac arrest with shockable rhythm (Ventricular fibrillation) (35 / 229)	19.7 [± 9.2] 122 [± 63.8]	29 [± 1] 192 [± 16.5]	53.3	83.7
Cardiac arrest with unshockable rhythm (Asytole) (31 / 180)	18.7 [± 4] 116 [± 23.5]	20.7 [± 5.5] 119 [± 46.5]	64.3	66.3
Haemodynamic unstable Tachycardia (27 / 186)	8.3 [± 1.5] 50 [± 8.7]	22 [± 1] 162 [± 3.6]	26.9	87.1
Haemodynamic unstable Bradycardia (17 / 109)	11.7 [± 2.1] 88 [± 10.1]	14.7 [± 0.6] 99 [± 10.4]	79.8	91.4

Sequence per scenario was *n* = 6, sequence per scenario + checklist yes/no was *n* = 3

To differentiate the effect of our checklist intervention from study participant’ pre-existing experience in handling medical crises, we integrated the average experience level of each team into the linear regression analysis model (see Supplementary Material). This did not change the outcome of the analysis, suggesting, that team experience was not a cofounding factor in the observed differences.

### Simulator data

The manikin used in this study is designed for CPR training. Its internal computer records CPR quality factors such as CPR depths and rate, thorax recoil and hand position during CPR, no-flow-time, ventilation volume and rate. Each of these CPR quality factors was displayed on the *Cardiac arrest* checklists. Despite their distinct display with colorful emphasis, analysis did not show any difference in these factors for teams with vs. teams without a checklist (Table 3). Consequently, we investigated determinants of CPR quality beyond the checklist. Unsurprisingly, participants’ experience in managing crises turned out to be the most dominant factor in influencing CPR quality (see Supplementary Material).

**Table 3:** Analysis of simulator data regarding CPR quality factors

	Mean value of all sequences without a checklist [± SD]	Mean value of all sequences with a checklist [± SD]	*p* value
Average CPR depth in mm (target: 50–60 mm)	48 [± 11]	45 [± 11]	0.311
Full chest recoil in relation to CPR time in % (target: > 90%)	42 [± 0.2]	38 [± 0.2]	0.749
Average CPR rate in compressions per minute (target: 100–120/min)	108 [± 9]	106 [± 11]	0.936
Correct hand position in relation to CPR time in % (target: > 90%)	91 [± 0.1]	94 [± 0.06]	0.81
No-flow-time in relation to CPR time in % (target: < 10%)	10.8 [± 0.07]	13 [± 0.08]	0.23
Average ventilation rate in breaths per minute (target: 6–8 breaths per minute)	9 [± 5]	9 [± 4]	0.689
Average ventilation volume in ml (target: 400–600 ml)	470 [± 147]	437 [± 91]	0.81

Mean value with standard deviation for all sequences a checklist was used (*n* = 12) or not (*n* = 12). *p* value in two-sided Mann–Whitney-*U*-test

### Questionnaire analysis

The response rate was 100%. An excerpt of the questionnaire analysis is presented in Table 4 (for full analysis, see Supplementary Material). Data show strong participant approval for the checklists and the scenarios, e.g., all participants rated the checklists as useful (56.25% agree strongly, 43.75% agree) and 96% disagreed with the statement that the checklist impeded the team in solving the scenario (62.5% disagree strongly, 33.5% disagree). All but one participant stated they would prefer the crisis management team to use a checklist if they themselves were a patient in a critical situation (62.5% agree strongly, 35.4% agree).

**Table 4:** Questionnaire analysis of scenarios with a checklist available (excerpt)

Survey Question	Agree strongly	Agree partly	Disagree partly	Disagree fully
I think our team’s performance profited from using the Checklist	13 (27.1%)	25 (52.1%)	9 (18.7%)	1 (2.1%)
I think the Checklist helped me to structure my actions	12 (25%)	27 (56.2%)	7 (14.6%)	2 (4.2%)
I would use the Checklist in reality	27 (56.2%)	17 (35.4%)	3 (6.3%)	1 (2.1%)
The Checklist impeded our performance	-	2 (4.2%)	16 (33.3)	30 (62.5%)
I think checklists in general are useful	27 (56.2%)	21 (43.8%)	–	–
I think a checklist for this scenario is useful	24 (50%)	23 (47.9%)	1 (2.1%)	–
I think for me personally checklists are useful	25 (52.1%)	23 (47.9%)	–	–
If I myself was a patient in the ER I’d like the personnel to use a checklist	30 (62.5%)	17 (35.4%)	1 (2.1%)	–

Numbers given in total count (*n* = 48), percentage in brackets

### Other results from video analysis

In five of six teams, the most experienced team member was elected team leader; whilst in one team, the most experience member was elected checklist reader.

In eleven of the twelve sequences, the checklist reader chose the checklist intended for the scenario. In one sequence, the “wrong” checklist was initially selected and the checklist reader changed to the correct checklist after 161 s. The checklist booklet was never given to another team member. In one sequence, the booklet was laid aside for 73 s.

## Discussion

In this study, the use of a crisis checklist was strongly associated with an increased adherence to standard procedures and successful completion of scenarios simulating medical emergencies. The study aimed to establish and test the effect of using checklists in a site specific context, for which the content and layout of the checklists were customized. A randomized cross-over design was chosen to minimize learning effects of study participants.

Crisis checklists have been successfully developed and employed in many medical settings, including intraoperative pediatric emergencies or evacuations from the operating room [34, 35]. While most crisis checklists are tailored towards perioperative situations or anesthesiology procedures, this study aimed to evaluate their use specifically for complex situations in emergency medicine that frequently occur in internal medicine, such as arrhythmias or cardiac arrest. Besides the complexity, such situations are characterized by a competent immediate need of action, but minimal time to evaluate the situation.

For this reason, we designed the lists to be short and easy to read with the opportunity to transfer the complexity into a feasible operating procedure. The increased performance associated with the use of a crisis checklist occurred despite the lack of prior instructions in their use to the participants. This suggests that one advantage of checklists is to serve as a useful ad hoc cognitive aid. In contrast to guideline charts or quick reference instruction cards, the checklists used in our study were specifically customized to the local setting. This has been found to be critical measure for successful implementation of checklists into clinical practice and may have contributed to the strong effect on performance [36]. We observed the highest benefit from checklist use in the hemodynamically unstable tachycardia sequence, an event that requires a complex technical approach. Site and equipment specific information provided by the checklist may facilitate the management of such cases. While our study was tailored to mimic the management of severe cardiac emergencies in an emergency room, it is possible to customize the checklists and procedures towards a different setting, such as inpatient wards. Checklists may have a stronger impact on the performance of less experienced teams. Integrating checklists and simulations may facilitate training of junior team members, and reinforce adherence to best practice care in training sessions [37, 38].

A possible disadvantage of the use of checklists is the potential to divert attention away from patient management in critical care situations. In our study, we did not find that the checklist had a distractive effect, neither in the self-assessment of participants nor in objective parameters, such as CPR quality. On the other hand, we also did not find CPR quality to benefit from checklist usage, consistent with the finding that intensive training is the most beneficial factor in this regard [39–41]. Following a checklist, however, does require additional resources. In our simulations, one team member communicated the checklist items to the team. Thus, checklists will be most efficiently implemented when enough staff is available that can devote sufficient attention to a checklist.

It has been pointed out that the most crucial aspect of implementing checklists into clinical routines is to adapt them to the local requirements of multidisciplinary teams [32, 42]. Improving the design and layout of the checklists may increase usability and, thus, lower the implementation burden, but iterative testing and constant revision are essential, all of which are resource intensive and require a concerted team effort [43].

One strong limitation of this study is that participants were medical students, and it is unclear how well our findings will translate to well trained and experienced medical professionals. However, 42% of participants were trained and worked as paramedics (a separate educational track to medical school in Germany) and, thus, had above-average experience in handling critical care situations. The statistical analysis did not find an association of experience level with checklist-dependent performance enhancement. Still, checklists cannot compensate knowledge or experience and, thus, cannot replace clinical decision making. Further studies, preferably with more teams, additional scenarios, and more experienced health care provider are needed to evaluate the utility of crisis checklists in clinical settings.

## Availability of data and material

Parts of the data underlying this study are available in aggregated and anonymized form in the supplementary material. Anonymized raw data can be made available from the authors upon request.

## Supplementary Information

Below is the link to the electronic supplementary material.

Supplementary file1 (PDF 6911 KB)

## Data Availability

Not applicable.
