# Supplementary material for: A simulation-based pilot study of crisis checklists in the emergency department
Source: Intern Emerg Med. 2021 Mar 9;16(8):2269–76. doi: 10.1007/s11739-021-02670-7 (PMC8563565; doi:10.1007/s11739-021-02670-7)
Supplement: Supplementary file 1 — Supplementary file1 (PDF 6911 KB) [file 11739_2021_2670_MOESM1_ESM.pdf]

Supplementary Appendix

1 Questionnaire

1.1 Original Questionnaire

UNIVERSITÄTS KLINIKUM FREIBURG

Questionnaire

Scenario:

Your opinions help us to improve our checklists in design and content, so we are very curious on what you have to say! This questionnaire is anonymous.

Please select the appropriate option below:

**Position**	Resident	Emergency physician	Nurse	Specialized nurse in emergency medicine	Paramedics	Student	Other
**Age**	20-24	25-30	30-34	35-40	>40		
**Sex**	F	M					
**Semester**	7	8	9	10	11	12	Practical Year
**Attended an ACLS class?**	Yes	No					

**Years in medical profession**	0	1-3	4-6	7-9	≥10
**Years in emergency medicine**	0	1-3	4-6	7-9	≥10

How often have you participated in the following medical scenarios?

**Cardiac Arrest**	0	1-3	4-6	7-9	≥10
**Unstable arrhythmia**	0	1-3	4-6	7-9	≥10

How experienced with above-named medical scenarios would you describe yourself?

Extremely experienced	Very experienced	Experienced	Moderately experienced	Little experienced	Unexperienced	I don't know

**Please complete following table if there was NO CHECKLIST in the scenario!**	**Agree strongly**	**Agree**	**Agree partly**	**Don't agree**
I think our team did a good job				
Considering the circumstances, I'm satisfied with the team's performance				
Considering the circumstances, I'm satisfied with my own performance				
The Scenario was realistic				
The Scenario was challenging				
I felt overstrained by the Scenario				
Please rate the Scenario on a scale from 1 to 4 (1 = very good, 4 = very bad)				

Please turn page!

**Please complete following table if there was A CHECKLIST in the scenario!**	**Agree strongly**	**Agree**	**Agree partly**	**Don't agree**
I think our team's performance profited from using the Checklist				
I think the Checklist helped our team to organise itself				
I felt more secure in the scenario using the Checklist				
I think the Checklist helped me structuring my actions				
I think I'd use the Checklist in reality				
Although having the Checklist, I didn't know how to approach the scenario				
The information provided by the Checklist weren't sufficient to solve our scenario				
The Checklist hampered our performance				
I think checklists in general are useful				
I think a checklist for this scenario is useful				
I think for me personally checklists are useful				
If I myself was a patient in the ER I'd like the personnel to use a checklist				
Please rate the Checklist on a scale from 1 to 4 (1 = very good, 4 = very bad)				
The Scenario was realistic				
The Scenario was challenging				
I felt overstrained by the Scenario				
Please rate the Scenario on a scale from 1 to 4 (1 = very good, 4 = very bad)				

**Please complete following table if you were the TEAMLEADER in the scenario!**	**Agree strongly**	**Agree**	**Agree partly**	**Don't agree**
The Checklist supported me in my tasks being the teamleader				
I felt my competence as the teamleader narrowed by the Checklist				

**Please complete following table if you were the CHECKLIST READER in the scenario!**	**Agree strongly**	**Agree**	**Agree partly**	**Don't agree**
The Checklist was easy to use				
The fond war clear and easy to read				
I think I felt less stressed by using the Checklist				
I didn't get along using the Checklist				
I think the Checklist was confusing				
The colours on the Checklist are irritating				
I think the information provided on the Checklist are to complex				
I had the feeling to hamper the team and the teamleader				

1.2 Questionnaire analysis

Table 1: Participants' characteristics

		Total Count	Percentage
Position	Student	24	100 %
	Paramedics	10	42 %
Semester	7th	5	21 %
	9th	10	42 %
	11th	3	12 %
	Practical Year	6	25 %
Age	20-24 years	9	38 %
	25-30 years	15	62 %
Sex	Female	11	46 %
	Male	13	54 %
Years of experience in medical profession	0	12	50 %
	1-3	6	25 %
	> 4	6	25 %
Years of experience in emergency medicine	0	14	58 %
	1-3	4	17 %
	> 4	6	25 %
Have you ever attended an ACLS class?	Yes	11	46 %
	No	13	54 %
How often have you participated in a cardiac arrest situation?	0	11	46 %
	1-3x	3	12 %
	4-6x	7	29 %
	> 7x	3	12 %
How often have you participated in an unstable arrythmia situation?	0 x	11	46 %
	1-3x	11	46 %
	> 4x	2	8 %
How experienced with above-named medical scenarios would you describe yourself?	Unexperienced	9	38 %
	Poorly experienced	11	46 %
	Moderately expierenced	3	12 %
	Experienced	1	4 %

Table 2: All scenarios (numbers given in total count, percentage in brackets)

Survey Question	Agree strongly	Agree	Agree Partly	Don't Agree
The Scenario was realistic	37 (38,5%)	52 (54,2%)	7 (7,3%)	-
The Scenario was challenging	44 (45,75%)	46 (48%)	6 (6,25%)	-
I felt overstrained by the Scenario	14 (14,6%)	54 (56,25%)	17(17,75%)	11 (11,4%)

Table 3: Scenario Rating

Scenario	Mean value on a scale from 1-4	Standard deviation
Cardiac arrest with shockable rhythm	1,41	± 0,49
Cardiac arrest with unshockable rhythm	1,7	± 0,45
Haemodynamic unstable Tachycardia	1,5	± 0,5
Haemodynamic unstable Bradycardia	1,41	± 0,49

Table 4: Scenarios with no checklist available (numbers given in total count, percentage in brackets)

Survey Question	Agree strongly	Agree	Agree Partly	Don't Agree
I think our team did a good job	7 (14,6%)	27 (56,2%)	14 (29,2%)	-
Considering the circumstances, I'm satisfied with the team's performance	10 (20,8%)	24 (50%)	14 (29,2%)	-
Considering the circumstances, I'm satisfied with my own performance	5 (10,4%)	16 (33,3%)	26 (54,2%)	1 (2,1%)

Table 5: Scenarios with a checklist available (numbers given in total count, percentage in brackets)

Survey Question	**Agree strongly**	**Agree**	**Agree Partly**	**Don't Agree**
I think our team's performance profited from using the Checklist	13 (27,1%)	25 (52,1%)	9 (18,7%)	1 (2,1%)
I think the Checklist helped our team to organise itself	13 (27%)	22 (44,8%)	11 (23%)	2 (4,2%)
I felt more secure in the scenario using the Checklist	9 (18,7%)	31 (64,6%)	7 (14,6%)	1 (2,1%)
I think the Checklist helped me structuring my actions	12 (25%)	27 (56,2%)	7 (14,6%)	2 (4,2%)
I think I'd use the Checklist in reality	27 (56,2%)	17 (35,4%)	3 (6,3%)	1 (2,1%)
Although having the Checklist, I didn't know how to approach the scenario	-	19 (39,5%)	19 (39,5%)	10 (20%)
The information provided by the Checklist weren't sufficient to solve our scenario	5 (10,4%)	13 (27,1%)	18 (37,5%)	12 (25%)
The Checklist hampered our performance	-	2 (4,2%)	16 (33,3)	30 (62,5%)
I think checklists in general are useful	27 (56,2%)	21 (43,8%)	-	-
I think a checklist for this scenario is useful	24 (50%)	23 (47,9%)	1 (2,1%)	-
I think for me personally checklists are useful	25 (52,1%)	23 (47,9%)	-	-
If I myself was a patient in the ER I'd like the personnel to use a checklist	30 (62,5%)	17 (35,4%)	1 (2,1%)	-

Table 6: Checklist Rating

Scenario	**Mean value on a scale from 1-4**	**Standard deviation**
Cardiac arrest with shockable rhythm	1,75	± 0,6
Cardiac arrest with unshockable rhythm	1,9	± 0,28
Haemodynamic unstable Tachycardia	1,5	± 0,65
Haemodynamic unstable Bradycardia	1,5	± 0,5

Table 7: Questions for the Checklist Reader (numbers given in total count, percentage in brackets)

Survey Question	**Agree strongly**	**Agree**	**Agree Partly**	**Don't Agree**
The Checklist was easy to use	3 (25%)	6 (50%)	3 (25%)	-
The fond war clear and easy to read	9 (75%)	3 (25%)	-	-
I think I felt less stressed by using the Checklist	2 (16,7%)	8 (66,6%)	2 (16,7%)	-
I didn't get along using the Checklist	-	5 (41,7%)	6 (50%)	1 (8,3%)
I think the Checklist was confusing	-	3 (25%)	7 (58,3%)	2 (16,7%)
The colours on the Checklist are irritating	-	-	3 (25%)	9 (75%)
I think the information provided on the Checklist are to complex	-	6 (50%)	3 (25%)	3 (25%)
I had the feeling to hamper the team and the teamleader	1 (8,3%)	2 (16,7%)	5 (41,7%)	4 (33,3%)

Table 8: Questions for the Teamleader (numbers given in total count, percentage in brackets)

Survey Question	**Agree strongly**	**Agree**	**Agree Partly**	**Don't Agree**
The Checklist supported me in my tasks being teamleader	5 (41,7%)	6 (50%)	1 (8,3%)	-
I felt my competence as teamleader narrowed by the Checklist	-	-	-	12 (100%)

2 Crisis Scenarios

2.1 Cardiac arrest with shockable rhythm: Ventricular fibrillation due to acute ST-elevation myocardial infarction of the anterior wall

Scenario setting and patient history: "You are the ER-team on duty tonight. Two nurses are occupied in the Trauma ER and the other doctor on call tonight is treating a patient with GI-bleeding. The ambulance arrives with a new patient and the paramedics present following patient history to you: 61year-old Mr. Frantz has gastric pain and dyspnea. About half an hour ago he vomited once and felt a piercing pain in the pit of his stomach. According to Mr. Frantz the pain isn't radiating. Mr. Frantz is very agitated and breathing heavily, he can't tell you anything about his medical history or medicaments in this situation. You have a look at his vital parameters: Heart rate 154 bpm, blood pressure 84/41 mmHg, peripheral oxygen saturation 88% with oxygen 6 l/min over mask, respiratory rate 32 per minute. Suddenly Mr. Frantz doesn't respond anymore. How do you proceed?"

Protocol: Change to sinus rhythm after 4th rhythm analysis if Epinephrine and Amiodarone were administered after 3rd defibrillation.

2.2 Cardiac arrest with unshockable rhythm: Asystole due to massive pulmonary embolism

Scenario setting and patient history: "You are the ER-team on duty this hectic Wednesday afternoon. Two nurses are occupied in the Trauma ER and the other doctor is treating a patient with GI-bleeding. Mrs. Müller, 55, came by herself two hours ago, her general physician doesn't have consultation hours today, so she came to your ER although she has never been to a hospital before. Normally she would never consult a doctor "just because of some coughing", but since this morning she is experiencing shortness of breath, in addition she feels a piercing thorax pain when breathing in. Mrs. Müller doesn't take any kind of medication although her general physician wants her to. While talking to her you notice she is sweating and her dyspnea is increasing fast, so you decide to lay her down and take her vital parameters: Heart rate 149 bpm, blood pressure 87/42 mmHg, peripheral oxygen saturation 85%, respiratory rate 34 per minute. Suddenly Mrs. Müller doesn't respond anymore. How do you proceed?"

Protocol: Change to sinus rhythm after 4th rhythm analysis if Epinephrine 1mg was administered 2 times throughout.

2.3 Haemodynamic unstable tachyarrhythmia: Ventricular tachycardia due to Hypokalaemia

Scenario setting and patient history: "You are the ER-team on duty tonight. Two nurses are occupied in the Trauma ER and the other doctor is treating a patient with GI-bleeding. The ambulance arrives with a new patient and the paramedics present following patient history to you: Mr. Müller, 84, resident in a senior's home, fell on his head 2 hours ago due to vertigo. For fours days he is experiencing heavy diarrhea. You want to have a look at his medication and medical history, but suddenly Mr. Müller seems to be on the verge of fainting and only responds with groaning, so you take his vital parameters: Heart rate 190 bpm, blood pressure 58/32 mmHg, peripheral oxygen saturation 86% with oxygen 8 l/min over mask, respiratory rate 33 per minute. How do you proceed?"

Protocol: Change to sinus rhythm after 3rd synchronised cardioversion with appropriate energy level plus administration of Amiodarone 300mg after 3rd cardioversion.

2.4 Haemodynamic unstable bradyarrhythmia: 3rd degree atrioventricular block due to hyperkalaemia

Scenario setting and patient history: "You are the ER-team on duty tonight. Two nurses are occupied in the Trauma ER and the other doctor is treating a patient with GI-bleeding. The ambulance arrives with a new patient and the paramedics present following patient history to you: 79year-old Mr. Schuster is feeling dizzy and increasingly

tired since this morning. He missed his dialysis appointment yesterday because he had to take care of his cancer-sick wife. Suddenly Mr. Müller seems to be on the verge of fainting and only responds with groaning, so you take his vital parameters: Heart rate 21 bpm, blood pressure 63/39 mmHg, peripheral oxygen saturation 88% with oxygen 6 l/min over mask, respiratory rate 28 per minute. How do you proceed?"

Protocol: Capture reached with following pacemaker settings: output pulse 70 pulses per minute and output current 130 mA.

3 Equipment

3.1 Equipment of the simulated ER

Object	Trading name	Corporation	Main Office
**Ultrasound**	CX50 Point-of-Care	Philips Healthcare Deutschland GmbH	Hamburg, Germany
**Stretcher**	Trauma Stretcher®	Stryker Deutschland GmbH und Co. KG	Duisburg, Germany
**Mannikin**	Resusci Anne QCPR®	Laerdal Medical GmbH	Puchheim, Germany
**Control-Tablet**	SimPad®Tablet	Laerdal Medical GmbH	Puchheim, Germany
**Patient monitor**		Laerdal Medical GmbH	Puchheim, Germany
**Mannikin connecting cable to defibrillator**		Laerdal Medical GmbH	Puchheim, Germany
**Defibrillator**	ZOLL® Serie R®	Zoll Medical GmbH	Cologne, Germany
**Ventilation bag with resevroir**	Ambu® SPUR® II	Ambu A/S	Ballerup, Denmark
**Ventilation mask**	Ambu® Einwegs-Gesichtsmaske PLUS	Ambu A/S	Ballerup, Denmark
**Disposal container**	Multi-Safe quick 1500	MSG medizinische Geräte, Handel und Service GmbH	Wuppertal, Germany
**Single use gloves, non-sterile**	Peha-soft® nitrile powderfree	Paul Hartmann AG	Heidenheim, Germany
**Sphygmomanometer**		Zoll Medical GmbH	Cologne, Germany
**Stethoskop**			
**1x Desinfection 250ml**	Softasept® N	B. Braun Melsungen AG	Melsungen, Germany
**Non-sterile swabs**	Zelletten®	Lohmann & Rauscher GmbH und Co. KG	Neuwied, Germany
**10x iv-cannula patch**	IV3000®	Smith&Nephew plc	London, GB
**10 x infusion system**	Intrafix® SafeSet	B. Braun Melsungen AG	Melsungen, Germany
**4 x infusion solution 500ml**	Jonosteril®	Fresenius Kabi GmbH	Bad Homburg, Germany
**Tourniquet**			
**iv-cannula 4x 16G / 18G / 20G**	Vasofix® Braunüle	B. Braun Melsungen AG	Melsungen, Germany
**5x Serum tube 9ml**	S-Monovette® Lithium-Heparin-Gel	Sarstedt AG & Co	Nümbrecht, Germany
**5x EDTA tube 9ml**	S-Monovette® K3 EDTA	Sarstedt AG & Co	Nümbrecht, Germany
**5x EDTA tube 2,7ml**	S-Monovette® K3 EDTA	Sarstedt AG & Co	Nümbrecht, Germany
**5x Citrat tube 3ml**	S-Monovette® Lithium-Heparin	Sarstedt AG & Co	Nümbrecht, Germany
**5x tube for cross-matching blood 9ml**	S-Monovette® Gerinnungsaktivator	Sarstedt AG & Co	Nümbrecht, Germany
**Monovette with Adapter for blood gas analysis**	safePICO® Probennehmer	Radiometer GmbH	Nümbrecht, Germany
**Adapter for S-Monovette**		Sarstedt AG & Co	Nümbrecht, Germany
**Laryngoscope**	F.O. Standard Laryngoskop	Heine Optotechnik GmbH und Co. KG	Herrsching, Germany
**Laryngoscope plates Macintosh size 3 / 4 / 5**		Heine Optotechnik GmbH und Co. KG	Herrsching, Germany
**Endotracheal tubes Murphy size 6,0 / 7,0 / 7,5 / 8,0**	RÜSCH® Super Safety Clear Flexiset	Teleflex Medical	Athlone, Ireland
**Fixation set for endotracheal tube**		VBM Medizintechnik GmbH	Sulz a.N., Germany

**Magill forceps**			
**Sterile gel**	Instillagel®	Farco-Pharma GmbH	Cologne, Germany
**Sterile guide wire Ch 12**		Teleflex Medical	Athlone, Ireland
**Wendl tubus Ch 22**		Mallinckrodt Pharmaceuticals	Staines-Upon-Thames, GB
**Guedel tubus size 3 / 4 / 5**	RÜSCH® Guedel / Oropharyngeal tubus	Teleflex Medical	Athlone, Ireland
**Laryngeal tube size 3 / 4 / 5**	LTS-D®	VBM Medizintechnik GmbH	Sulz a.N., Germany
**1 CO2-Cuvet**	Respironics® CAPNOSTAT® 5	Zoll Medical GmbH	Cologne, Germany

3.2 Medicaments, cannulas, syringes

Trading name + volume + dosage	Active substance	Corporation	Main Office
**5 vials of Atropin 0,5mg / ml**	Atropinesulfate	B. Braun Melsungen AG	Melsungen, Germany
**3 vials of Cordarex® 150mg / 3ml**	Amiodarone-hydrochloride	Sanofi-Aventis Deutschland GmbH	Frankfurt, Germany
**5 vials of Adrekar® 6mg / 2ml**	Adenosine	Sanofi-Aventis Deutschland GmbH	Frankfurt, Germany
**5 Lifeshield Glass Abboject ready-to-fill-syringe 1mg 0,1mg / ml**	Epinephrine	Hospira Incorporated	Lake Forest, Illinois, USA
**3 vials of Ketanest® S 125mg / 5ml**	S-Ketamine	Pfizer Manufacturing Deutschland GmbH	Freiburg, Deutschland
**3 vials of Dormicum® 15mg / 3ml**	Midazolam	Roche Pharma AG	Grenzach-Whylen, Germany
**3 vials of Dipidolor® 7,5mg / ml**	Pirtramid	Janssen-Cilag GmbH	Neuss, Germany
**3 vials of Propofol 1 % Mct 200mg / 20ml**		Fresenius Kabi GmbH	Bad Homburg, Germany
**5 vials of NaCL 0,9% 10ml**	Sodium chloride 0,9%	B. Braun Melsungen AG	Melsungen, Germany
**Syringe Inject® Solo, 5x 2ml / 5ml / 10ml**		B. Braun Melsungen AG	Melsungen, Germany
**Cannula Sterican®, 5x Größe 2 / 12 / 20**		B. Braun Melsungen AG	Melsungen, Germany

4 Key Processes

4.1 Cardiac arrest with shockable rhythm

Key Process	Point value	Timing	Point value for timing	Supporting literature
Teamleader announced	1	Within 25s	2	6, 17, 23
Chest compressions initiated	10	Within 40s	12	2, 7, 13, 14, 33
Bag-ventilation initiated	3	Within 1min	3	35
Defibrillator connected	10	Within 1min	12	11, 24, 36, 39
1st rhythm analysis	5	Within 1:15min	8	11, 24, 36, 39
1st shock administered	5	15s after 1st rhythm analysis	12	1, 10, 27, 32, 40
Airway secured	5	Within 3min	8	21
i.v.-line checked	1			35
2nd rhythm analysis	5	2min after 1st rhythm analysis ± 20s	8	
2nd shock administered	5	15s after 2nd rhythm analysis	12	
3rd rhythm analysis	5	2min after 2nd rhythm analysis ± 20s	8	
3rd shock administered	5	15s after 3rd rhythm analysis	12	
Adrenaline 1mg administered	2	After 3rd shock	2	15, 18, 29
Amiodarone 300mg administered	2	After 3rd shock	2	9, 22, 30
Reversible causes discussed	8			35, 38
FEEL	5	10s	5	4, 16
4th rhythm analysis	5	2min after 3rd rhythm analysis ± 20s	8	
ROSC reached	15			
BGA	8			35, 38
Diagnosis	10			35, 38

4.2 Cardiac arrest with unshockable rhythm

Key Process	Point value	Timing	Point value for timing	Supporting literature
Teamleader announced	1	Within 25s	2	6, 17, 23
Chest compressions initiated	10	Within 40s	12	2, 7, 13, 14, 33
Bag-ventilation initiated	3	Within 1min	3	35
Defibrillator connected	10	Within 1min	12	11, 24, 36, 39
1st rhythm analysis	5	Within 1:15min	8	11, 24, 36, 39
1st administration of adrenaline 1mg	2	Within 40s after 1st rhythm analysis	2	8, 18, 29
Airway secured	5	Within 3min	8	21
i.v.-line checked	1			35
2nd rhythm analysis	5	2min after 1st rhythm analysis ± 20s	8	
Technical Re-Check if asystole	2			35
3rd rhythm analysis	5	2min after 2nd rhythm analysis ± 20s	8	
2nd administration of adrenaline 1mg	2	3-5min after 1st adrenaline administration	2	
Reversible causes discussed	8			35, 38
FEEL	5	10s	5	4, 12, 16, 28, 37
4th rhythm analysis	5	2min after 3rd rhythm analysis ± 20s	8	
ROSC reached	15			
BGA	8			35, 38
Diagnosis	10			35, 38

4.3 Haemodynamic unstable tachyarrhythmia

Key Process	Point value	Timing	Point value for timing	Supporting literature
Teamleader announced	1	Within 25s	2	6, 17, 23
Oxygen administration initiated	2			
Defibrillator connected	10	Within 1:30min	12	
i.v.-line checked	1			35
Analgesia + Sedation administered	8			35
Sedation checked	5			
1st synchronisation	8			25
Energy-level at 120J	2			19, 20, 31
1st shock	10	Within 3min	12	25
2nd synchronisation	8			
Energy-level at 150J	2			
2nd shock	10	Max. 1min after 1st shock	12	
3rd synchronisation	8			
Energy-level at 200J	2			
3rd shock	10	Max. 1min after 2nd shock	12	
Amiodarone 300mg administered	2	After 3rd shock	2	35
Reversible causes discussed	8			35, 38
Haemodynamic stability	15			
BGA	8			35, 38
Echocardiography	5			35, 38
Diagnosis	10			35, 38

4.4 Haemodynamic unstable bradyarrhythmia

Key Process	Point value	Timing	Point value for timing	Supporting literature
Teamleader announced	1	Within 25s	2	6, 17, 23
Oxygen administration initiated	2			
Atropine 0,5mg administered				3, 5, 34
Defibrillator connected	10	Within 3min	12	
i.v.-line checked	1			35
Analgesia + Sedation administered	8			35
Sedation checked	5			
Pacemaker mode	8			
Pacing Stimuli	2			
Capture	15	Innerhalb 6min	8	26, 35
Reversible causes discussed	8			35, 38
Haemodynamic stability	15			
BGA	8			35, 38
Echocardiography	5			35, 38
Diagnosis	10			35, 38

IV.V Supporting literature

1 Auble TE, Menegazzi JJ, Paris PM (1995). *Effect of out-of hospital defibrillation by basic life support providers on cardiac arrest mortality: a metaanalysis.* Annals of Emergency Medicine, 25(5):642-8.

2 Baker PW, Conway J, Cotton C, Ashby DT, Smyth J, Woodman RJ, Grantham H, Clinical Investigators. *Defibrillation or cardiopulmonary resuscitation first for patients with out-of-hospital cardiac arrests found by paramedics to be in ventricular fibrillation? A randomised control trial.* Resuscitation 2008; 79(3):424-31.

3 Brady WJ, Swart G, DeBehnke DJ, Ma OJ, Aufderheide TP (1999). *The efficacy of atropine in the treatment of hemodynamically unstable bradycardia and atrioventricular block: prehospital and emergency department considerations.* Resuscitation 1999; 41(1):47-55.

4 Breitkreutz R, Price S, Steiger HV, Seeger FH, Ilper H, Ackermann H, Rudolph M, Uddin S, Weigand MA, Müller E, Walcher F; Emergency Ultrasound Working Group of the Johann Wolfgang Goethe-University Hospital, Frankfurt am Main (2010). *Focused echocardiographic evaluation in life support and peri-resuscitation of emergency patients: A prospective trial.* Resuscitation, 81(11):1527-33.

5 Chadda KD, Lichstein E, Gupta PK, Kourtesis P (1977). *Effects of atropine in patients with bradyarrhythmia complicating myocardial infarction: usefulness of an optimum dose for overdrive.* The American Journal of Medicine. 63(4):503-10.

6 Cooper S, Wakelam A. *Leadership of resuscitation teams: "Lighthouse Leadership".* Resuscitation 1999; 42(1):27-45.

7 Cummins RO, Eisenberg MS, Hallstrom AP, Litwin PE (1985). *Survival of out-of-hospital cardiac arrest with early initiation of cardiopulmonary resuscitation.* American Journal of Emergency Medicine, 3(2):114-9.

8 Donnino MW, Salciccioli JD, Howell MD, Cocchi MN, Giberson B, Berg K, Gautam S, Callaway C; American Heart Association's Get With The Guidelines-Resuscitation Investigators. *Time to administration of epinephrine and outcome after in-hospital cardiac arrest with non-shockable rhythms: retrospective analysis of large in-hospital data registry.* Br Med J 2014; 348:g3028.

9 Dorian P, Cass D, Schwartz B, Cooper R, Gelaznikas E, Barr A. *Amiodarone as compared with lidocaine for shock-resistant ventricular fibrillation.* N Eng J Med 2002; 346(12):884-90.

10 Eisenberg MS, Copass MK, Hallstrom AP, Blake B, Bergner L, Short FA, Cobb LA. *Treatment of out-of-hospital cardiac arrests with rapid defibrillation by emergency medical technicians.* N Eng J Med 1980; 302(25):1379-83.

11 Eisenberg MS, Hallstrom AP, Copass MK, Bergner L, Short F, Pierce J (1984). *Treatment of ventricular fibrillation: emergency medical technician defibrillation and paramedic services.* Journal of the American Medical Association, 251(13):1723-6.

12 Flato UA, Paiva EF, Carballo MT, Buehler AM, Marco R, Timerman A. *Echocardiography for prognostication during the resuscitation of intensive care unit patients with non-shockable rhythm cardiac arrest.* Resuscitation 2015; 92:1-6.

13 Gallagher EJ, Lombardi G, Gennis P (1995). *Effectiveness of bystander cardiopulmonary resuscitation and survival following out-of-hospital cardiac arrest.* Journal of the American Medical Association, 274(24):1922-5.

14 Hasselqvist-Ax I, Riva G, Herlitz J, Rosenqvist M, Hollenberg J, Nordberg P, Ringh M, Jonsson M, Axelsson C, Lindqvist J, Karlsson T, Svensson L. *Early cardiopulmonary resuscitation in out-of-hospital cardiac arrest.* N Eng J Med 2015; 372(24):2307-15.

15 Herlitz J, Ekstrom L, Wennerblom B, Axelsson A, Bang A, Holmberg A. *Adrenaline in out-of-hospital ventricular fibrillation. Does it make any difference?* Resuscitation 1995; 29(3):195-201.

16 Hernandez C, Shuler K, Hannan H, Sonyika C, Likourezos A, Marshall J. *C.A.U.S.E.: Cardiac arrest ultra-sound exam – a better approach to managing patients in primary non-arrhythmogenic cardiac arrest.* Resuscitation 2008; 76(2):198-206.

17 Hunziker S, Johansson AC, Tschan F, Semmer NK, Rock L, Howell MD, Marsch S (2011). *Teamwork and leadership in cardiopulmonary resuscitation.* Journal of the American College of Cardiology, 57(24):2381-8.

18 Jacobs IG, Finn JC, Jelinek GA, Oxer HF, Thompson PL. *Effect of adrenaline on survival in out-of-hospital cardiac arrest: a randomised double-blind placebo-controlled trial.* Resuscitation 2011; 82(9):1138-43.

19 Kerber RE, Kienzle MG, Olshansky B, Waldo AL, Wilber D, Carlson MD, Aschoff AM, Birger S, Fugatt L, Walsh S, Rockwell R, Charbonnier F. *Ventricular tachycardia rate and morphology determine energy and current requirements for transthoracic cardioversion.* Circulation 1992; 85(1):158-63.

20 Kerber RE, Martins JB, Kienzle MG, Constantin L, Olshansky B, Hopson R, Charbonnier F. *Energy, current, and success in defibrillation and cardioversion: clinical studies using an automated impedance-based method of energy adjustment.* Circulation 1988; 77(5):1038-46.

21 Kramer-Johansen J, Wik L, Steen PA. *Advanced cardiac life support before and after tracheal intubation – direct measurements of quality.* Resuscitation 2006; 68(1):61-9.

22 Kudenchuk PJ, Cobb LA, Copass MK, Cummins RO, Doherty AM, Fahrenbruch CE, Hallstrom AP, Murray WA, Olsufka M, Walsh T. *Amiodarone for resuscitation after out-of-hospital cardiac arrest due to ventricular fibrillation.* N Eng J Med 1999; 341(12):871-8.

23 Künzle B, Kolbe M, Grote G (2010). *Ensuring patient safety through effective leadership behaviour: a literature review.* Safety Science, 48(1):1-17.

24 Larsen MP, Eisenberg MS, Cummins RO, Hallstrom AP (1993). *Predicting survival from out-of-hospital cardiac arrest: a graphic model.* Annals of Emergency Medicine, 22(11):1652-8.

25 Lown B (1967). *Electrical reversion of cardiac arrhythmias*. British Heart Journal. 29(4):469-89.

26 Morrison LJ, Long J, Vermeulen M, Schwartz B, Sawadsky B, Frank J, Cameron B, Burgess R, Shield J, Bagley P, Mausz V, Brewer JE, Dorian P. *A randomized controlled feasibility trial comparing safety and effectiveness of prehospital pacing versus conventional treatment: 'PrePACE.'* Resuscitation 2008; 76(3):341-9.

27 Nichol G, Stiell IG, Laupacis A, Pham B, De Maio VJ, Wells GA (1999). *A cumulative meta-analysis of the effectiveness of defibrillator-capable emergency medical services for victims of out-of-hospital cardiac arrest.* Annals of Emergency Medicine, 34(4 Part 1): 517-25.

28 Niendorff DF, Rassias AJ, Palac R, Beach ML, Costa S, Greenberg M. *Rapid cardiac ultrasound of inpatients suffering PEA arrest performed by nonexpert sonographers.* Resuscitation 2005; 67(1):81-7.

29 Olasveengen TM, Sunde K, Brunborg C, Thowsen J, Steen PA, Wik L (2009). *Intravenous drug administration during out-of-hospital cardiac arrest: a randomized trial.* Journal oft he American Medical Association, 302(20):2222-9.

30 Petrovic T, Adnet F, Lapandry C (1998). *Successful resuscitation of ventricular fibrillation after low-dose amiodarone.* Annals of Emergency Medicine, 32(4):518-9.

31 Reisinger J, Gstrein C, Winter T, Zeindlhofer E, Hollinger K, Mori M, Schiller A, Winter A, Geiger H, Siostrzonek P (2010). *Optimization of initial energy for cardioversion of atrial tachyarrhythmias with biphasic shocks.* American Journal of Emergency Medicine, 28(2):159-65.

32 Sanna T, La Torre G, de Waure C, Scapigliati A, Ricciardi W, Dello Russo A, Pelargonio G, Casella M, Bellocci F. *Cardiopulmonary resuscitation alone vs. cardiopulmonary resuscitation plus automated external defibrillator use by non-healthcare professionals: a meta-analysis on 1583 cases of out-of-hospital cardiac arrest.* Resuscitation 2008; 76(2):226-32.

33 Sasson C, Rogers MA, Dahl J, Kellermann AL (2010). *Predictors of survival from out-of-hospital cardiac arrest: a systematic review and meta-analysis.* Circulation: Cardiovascular Quality & Outcomes, 3(1):63-81.

34 Smith I, Monk TG, White PF (1994). *Comparison of transesophageal atrial pacing with anticholinergic drugs for the treatment of intraoperative bradycardia.* Anesthesia & Analgesia. 78(2):245-52.

35 Soar J, Nolan JP, Böttiger BW, Perkins GD, Lott C, Carli P, Pellis T, Sandroni C, Skrifvars MB, Smith GB, Sunde K, Deakin CD, on behalf of the Adult advanced life support section Collaborator. *European*

Resuscitation Council Guidelines for Resuscitation 2015: Section 3. Adult advanced life support. Resuscitation 2015; 95:100-147.

36 Stiell IG, Nichol G, Leroux BG, Rea TD, Ornato JP, Powell J, Christenson J, Callaway CW, Kudenchuk PJ, Aufderheide TP, Idris AH, Daya MR, Wang HE, Morrison LJ, Davis D, Andrusiek D, Stephens S, Cheskes S, Schmicker RH, Fowler R, Vaillancourt C, Hostler D, Zive D, Pirrallo RG, Vilke GM, Sopko G, Weisfeldt M; ROC Investigators. *Early versus later rhythm analysis in patients with out-of-hospital cardiac arrest.* N Eng J Med 2011; 365(9):787-97.

37 Tayal VS, Kline JA. *Emergency echocardiography to detect pericardial effusion in patients in PEA and near-PEA states.* Resuscitation 2003; 59(3):315-8.

38 Truhlář A, Deakin CD, Soar J, Khalifa GE, Alfonzo A, Bierens JJ, Brattebø G, Brugger H, Dunning J, Hunyadi-Antičević S, Koster RW, Lockey DJ, Lott C, Paal P, Perkins GD, Sandroni C, Thies KC, Zideman DA, Nolan JP; Cardiac arrest in special circumstances section Collaborators. *European Resuscitation Council Guidelines for Resuscitation 2015: Section 4. Cardiac arrest in special circumstances.* Resuscitation 2015; 95:148-201.

39 Valenzuela TD, Roe DJ, Cretin S, Spaite DW, Larsen MP. *Estimating effectiveness of cardiac arrest interventions: a Logistic regression survival model.* Circulation 1997; 96(10):3308-13.

40 Weaver WD, Copass MK, Bufi D, Ray R, Hallstrom AP, Cobb LA. *Improved neurologic recovery and survival after early defibrillation.* Circulation 1984; 69(5):943-8.

5 Statistical analysis

5.1 Primary Endpoint

Table 1 Contingency Tables

Overall

PrimOutcome	Checklist	
	0	1
0	10	4
1	2	8

Odds Ratio = 10 (p = 0.03607 with Fisher's exact test)

Scenario Ventricular fibrillation

PrimOutcome	Checklist	
	0	1
0	3	1
1	0	2

Odds Ratio = ∞

Scenario Asystole

PrimOutcome	Checkliste	
	0	1
0	3	2
1	0	1

Odds Ratio = ∞

Scenario Unstable Tachycardia

PrimOutcome	Checkliste	
	0	1
0	3	1
1	0	2

Odds Ratio = ∞

Scenario Unstable Brandycardia

PrimOutcome	Checkliste	
	0	1
0	1	0
1	2	3

Odds Ratio = ∞

GEE Results:

Odds Ratio (CI)	z	p
10.0<br>[1.12; 89.38]	2.06	0.02

5.2 Secondary endpoint analysis

Table 1 Bivariable regression analysis

	Estimate	Std. Error	t value	Pr(>|t|)
(Intercept)	0.55449	0.08510	6.516	3.06e-6**
Checklist	0.26074	0.07612	3.426	0.00284 **
(Scenario)2	-0.03208	0.10764	-0.298	0.76890
(Scenario)3	-0.11497	0.10764	-1.068	0.29888
(Scenario)4	0.17141	0.10764	1.592	0.12781

Table 2: Interactive effects analyzed by bivariable regression analysis

	Estimate	Std. Error	t value	Pr(>|t|)
(Intercept)	0.53275	0.08718	6.111	1.5e-5 ***
Checklist	0.30422	0.12329	2.468	0.0253 *
(Scenario)2	0.10984	0.12329	0.891	0.3862
(Scenario)3	-0.26393	0.12329	-2.141	0.0480 *
(Scenario)4	0.26541	0.12329	2.153	0.0469 *
Checklist: (Scenario)2	-0.28385	0.17436	-1.628	0.1231
Checklist: (Scenario)3	0.29793	0.17436	1.709	0.1068
Checklist: (Scenario)4	-0.18801	0.17436	-1.078	0.2969

Table 3: Random effect analysis

Groups	Name	Variance	Std. Dev.	Corr
teamID	(Intercept)	0.04526	0.2127	
	Checklist	0.02726	0.1651	-1.00
Residual		0.02028	0.1424	
Number of obs: 24, groups: teamID, 6				

Table 4: Fixed effects

	Estimate	Std. Error	t value
(Intercept)	0.56058	0.09609	5.834
Checklist	0.26074	0.08901	2.929

Table 5: Logistic regression model of the primary outcome taking the competence of participants into account

	Estimate	Std. Error	Z value	Pr(>|z|)
(Intercept)	-3.273	1.868	-1.752	0.0798
Checklist	2.445	1.047	2.334	0.0196 *
as.numeric(competence)	4.258	4.107	1.037	0.2998

Table 6: Linear regression model of the secondary outcome taking the competence of participants into account:

	Estimate	Std. Error	Z value	Pr(>|t|)
(Intercept)	0.40595	0.13523	3.002	0.00679 **
Checklist	0.26074	0.08242	3.164	0.00468 **
as.numeric(competence)	0.41933	0.33092	1.267	0.21897

6 Simulator Data

Table 1: Analysis of Simulator data regarding CPR quality factors in team comparison. 2 of the 6 teams in this study were rated as “unexperienced” by questionnaire analysis and 4 teams were rated as “experienced”. Mean value with standard deviation for all CPR sequences completed by unexperienced teams (n = 4) and experienced teams (n = 8). P-value in one-sided student-t-test plus calculated degree of freedom (df) disclosed.

	**Mean value of all sequences completed by unexperienced teams [± SD]**	**Mean value of all sequences completed by experienced teams [± SD]**	**p-value**
Average CPR depth in mm (target: 50-60mm)	32,5 [± 7]	54 [± 3]	0.0056 [df = 3]
Full chest recoil in relation to CPR time in % (target: >90%)	37 [± 0.3]	42 [± 0.1]	0.401 [df = 3]
Average CPR rate in compressions per minute (target: 100-120/min)	103 [± 11]	109 [± 10]	0.214 [df = 5]
Correct hand position in relation to CPR time in % (target: >90%)	83 [+ 0.09]	97 [± 0.02]	0.04 [df = 3]
No-flow-time in relation to CPR time in % (target: <10%)	19,51 [± 0.08]	8.08 [± 0.02]	0.047 [df = 3]
Average ventilation rate in breaths per minute (target: 6-8 breaths per minute)	4 [± 1.5]	12 [± 1.8]	0.0002 [df = 8]
Average ventilation volume in ml (target: 400-600ml)	575 [± 117]	393 [± 61]	0.045 [df = 3]

7 The original checklists with English translation

7.1 The Frontpage: Emergency Room Checklists

Schockraum - Checklisten

UNIVERSITÄTS KLINIKUM FREIBURG

Fall	INDEX
bewusstloser Patient ohne Atmung<br>Reanimation - VF / VT	1
bewusstloser Patient ohne Atmung<br>Reanimation - Asystolie / PEA	2
HF >100/min + Schock / Vigilanzänderung / akute Herzinsuffizienz<br>Instabile Tachykardie mit Puls	3
HF <60/min + Schock / Vigilanzänderung / akute Herzinsuffizienz<br>Instabile Bradykardie mit Puls	4

>> Checklisten im Schockraum belassen! <<

Revised February 2016

Based on the OR-Crisis-Checklists at www.projectcheck.org/crisis.

Die Verantwortung für die Umsetzung der Maßnahmen und den Gebrauch der Checklisten liegt beim Anwender

>>Leave Checklists in ER<<

The responsibility for interpretation and execution of he information provided lies with the operator.

Case:

1. Unconscious Patient without respiration: Cardiac Arrest – VF / VT

2. Unconscious Patient without respiration: Cardiac Arrest – Asystole / PEA

3. Heart rate > 100/min + haemodynamic shock / altered vigilance / acute heart failure: Haemodynamic unstable Tachycardia with pulse

4. Heart rate < 60/min + haemodynamic shock / altered vigilance / acute heart failure: Haemodynamic unstable Bradycardia with pulse

7.2 Cardiac Arrest ► VF/ pulseless VT

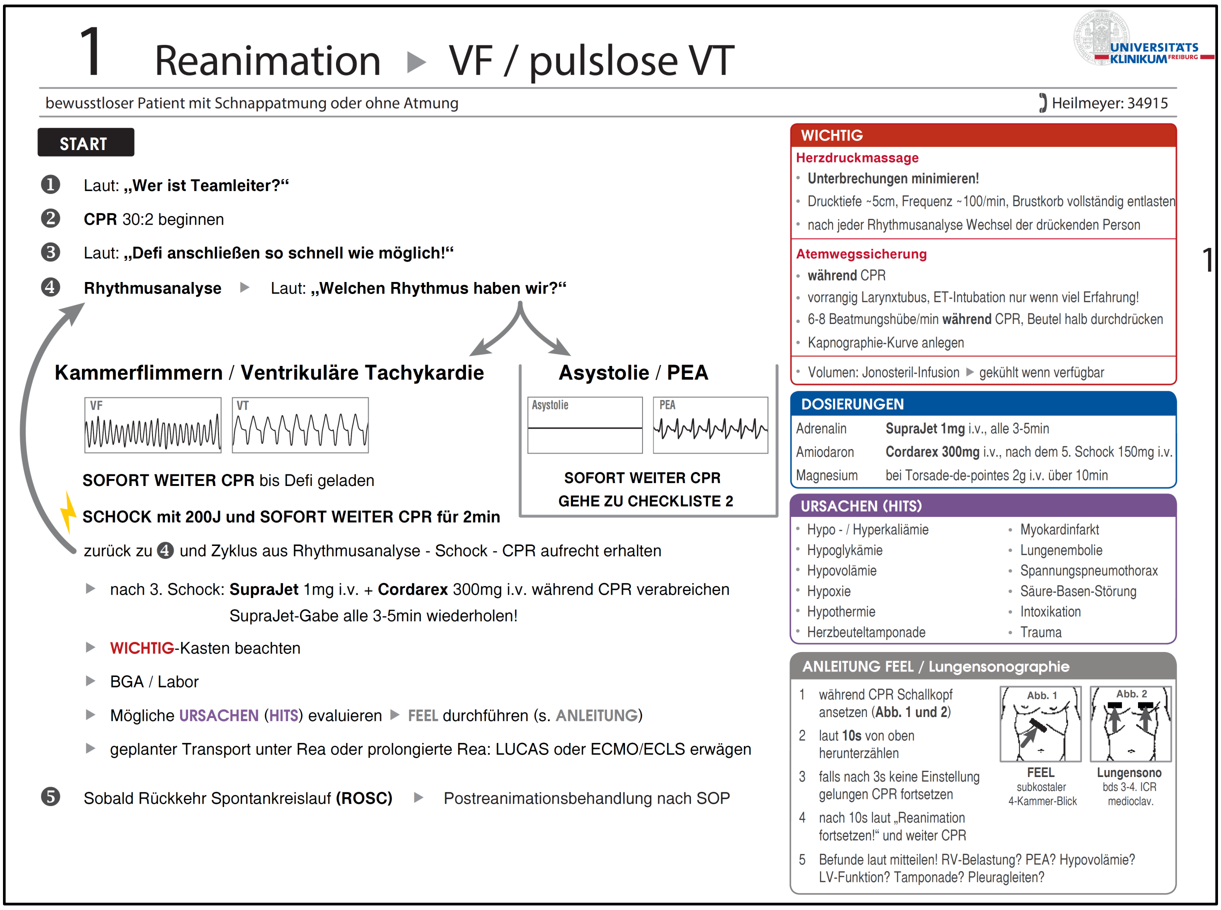

1 Reanimation ► VF / pulslose VT

bewusstloser Patient mit Schnappatmung oder ohne Atmung — Heilmeyer: 34915

START

❶ Laut: **„Wer ist Teamleiter?“**

❷ **CPR** 30:2 beginnen

❸ Laut: **„Defi anschließen so schnell wie möglich!“**

❹ **Rhythmusanalyse** ► Laut: **„Welchen Rhythmus haben wir?“**

Kammerflimmern / Ventrikuläre Tachykardie

VF — VT

SOFORT WEITER CPR bis Defi geladen

SCHOCK mit 200J und SOFORT WEITER CPR für 2min

Asystolie / PEA

Asystolie — PEA

SOFORT WEITER CPR

GEHE ZU CHECKLISTE 2

zurück zu ❹ und Zyklus aus Rhythmusanalyse - Schock - CPR aufrecht erhalten

► nach 3. Schock: **SupraJet** 1mg i.v. + **Cordarex** 300mg i.v. während CPR verabreichen
SupraJet-Gabe alle 3-5min wiederholen!

► WICHTIG-Kasten beachten

► BGA / Labor

► Mögliche URSACHEN (HITS) evaluieren ► FEEL durchführen (s. ANLEITUNG)

► geplanter Transport unter Rea oder prolongierte Rea: LUCAS oder ECMO/ECLS erwägen

❺ Sobald Rückkehr Spontankreislauf **(ROSC)** ► Postreanimationsbehandlung nach SOP

WICHTIG

Herzdruckmassage
- **Unterbrechungen minimieren!**
- Drucktiefe ~5cm, Frequenz ~100/min, Brustkorb vollständig entlasten
- nach jeder Rhythmusanalyse Wechsel der drückenden Person

Atemwegssicherung
- **während** CPR
- vorrangig Larynxtubus, ET-Intubation nur wenn viel Erfahrung!
- 6-8 Beatmungshübe/min **während** CPR, Beutel halb durchdrücken
- Kapnographie-Kurve anlegen

- Volumen: Jonosteril-Infusion ► gekühlt wenn verfügbar

DOSIERUNGEN

Adrenalin	**SupraJet 1mg** i.v., alle 3-5min
Amiodaron	**Cordarex 300mg** i.v., nach dem 5. Schock 150mg i.v.
Magnesium	bei Torsade-de-pointes 2g i.v. über 10min

URSACHEN (HITS)

Hypo - / Hyperkaliämie	Myokardinfarkt
Hypoglykämie	Lungenembolie
Hypovolämie	Spannungspneumothorax
Hypoxie	Säure-Basen-Störung
Hypothermie	Intoxikation
Herzbeuteltamponade	Trauma

ANLEITUNG FEEL / Lungensonographie

1 während CPR Schallkopf ansetzen (**Abb. 1 und 2**)
2 laut **10s** von oben herunterzählen
3 falls nach 3s keine Einstellung gelungen CPR fortsetzen
4 nach 10s laut „Reanimation fortsetzen!“ und weiter CPR
5 Befunde laut mitteilen! RV-Belastung? PEA? Hypovolämie? LV-Funktion? Tamponade? Pleuragleiten?

Abb. 1 — **FEEL** subkostaler 4-Kammer-Blick
Abb. 2 — **Lungensono** bds 3-4. ICR medioclav.

Unconscious patient with agonal or no respiration ☏ ICU

START

❶ *Loud: “Who is team leader?”*

❷ *Start CPR 30:2*

❸ *Loud: “Connect Defibrillator as fast as possible!”*

❹ *Rhythm analysis ► Loud: “What rhythm do we have?”*

→ *Ventricular Fibrillation / Ventricular Tachycardia: Resume CPR immediately until Defi is charged*

→ *Asystole / PEA: Resume CPR immediately, got to Checklist 2*

[redacted]*hock with 200J and resume CPR immediately for 2 minutes*

Go back to ❹ and maintain rhythm analysis – shock – CPR cycle

► *after 3rd shock: Administer SupraJet 1mg iv + Cordarex 300mg iv whilst CPR*

Administer SupraJet every 3-5min

► *mind Essential box*

► *blood gas analysis / blood sample*

► *evaluate possible Causes (Hs / Ts) ► perform FEEL (Instructions)*

► *transportation under CPR or prolonged CPR ► consider LUCAS or ECMO/ECLS*

❺ *ROSC ► see post-reanimation protocol*

ESSENTIAL

Cadiac massage

- *Reduce interruptions*
- *~5cm Compression depths, ~100 compressions per min, full chest recoil*
- *Change person performing cardiac massage after every rhythm analysis*

Airway management

- *During CPR*
- *Preferably laryngeal tube, endotracheal by experienced personnel!*
- *6-8 breaths per minute during CPR*
- *Apply capnography*

- *Administer balanced eletrolyte solutions (e.g. Jonosteril®), preferably cooled*

DRUGS & DOSING

Adrenaline	*SupraJet® 1mg iv, every 3-5min*
Amiodarone	*Cordarex® 300mg iv, after 5th shock 150mg iv*
Magnesium	*in case of Torsade-de-pointes 2g iv over 10min*

Reversible CAUSES (Hs & Ts)

- *Hypo- / Hyperkalaemia*
- *Hypoglycaemia*
- *Hypovolaemia*
- *Hypoxia*
- *Hypothermia*
- *Hydrogen Ion (acidosis or alkalosis)*
- *Thrombosis, coronary*
- *Thrombosis, pulmonary*
- *Tension pneumothorax*
- *Tamponade, cardiac*
- *Toxins*
- *Trauma*

INSTRUCTIONS: FEEL / Lung ultrasound

1. *Position ultrasound probe during CPR*
2. *Count down from 10*
3. *If after 3sec no accurate imaging resume CPR*
4. *Resume CPR after 10sec*
5. *Evaluate findings: RV strain? PEA? Hypovolaemia? LV function? Tamponade? Pleural sliding?*

7.3 Cardiac Arrest ► Asystole / PEA

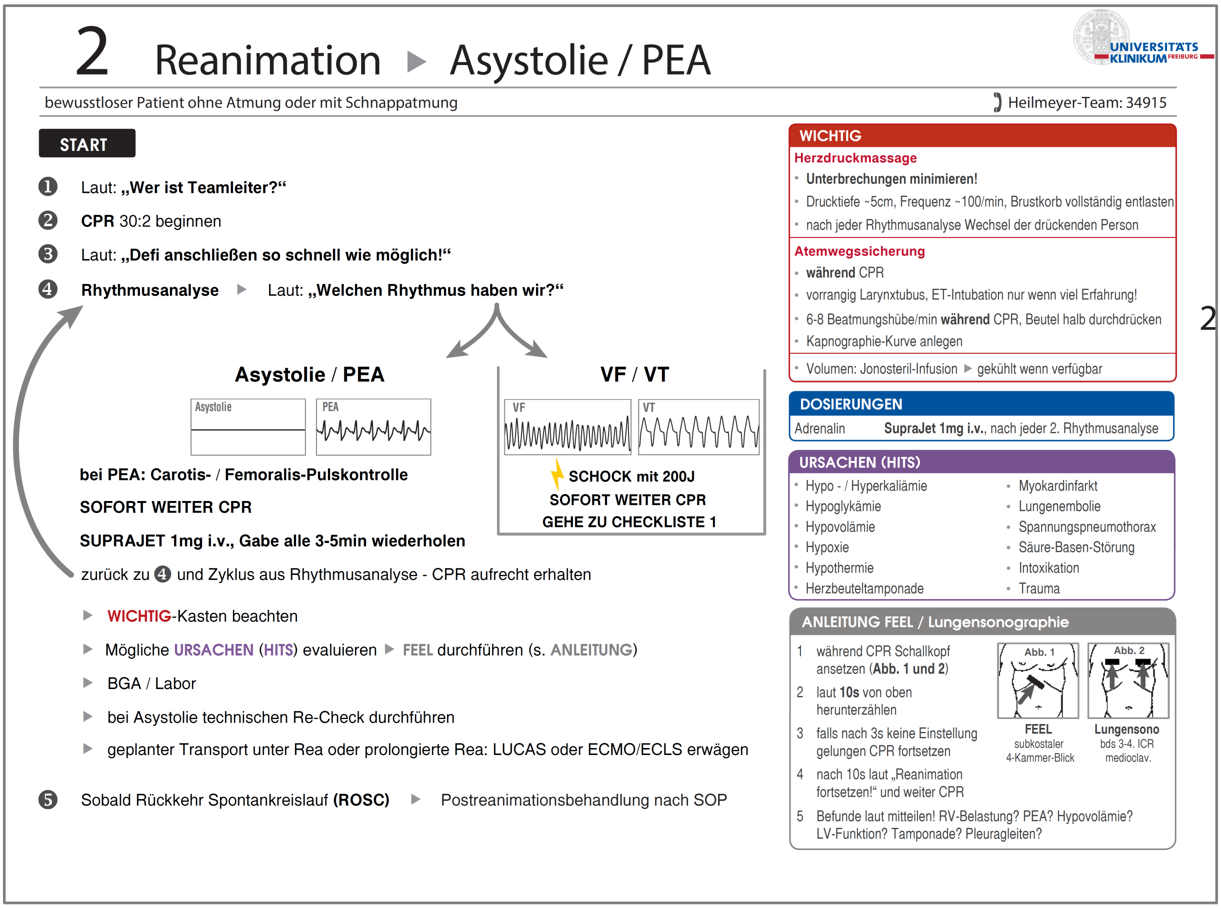

Unconscious patient with agonal or no respiration ☎ ICU

START

❶ Loud: "Who is team leader?"

❷ Start CPR 30:2

❸ Loud: "Connect Defibrillator as fast as possible!"

❹ Rhythm analysis ► Loud: "What rhythm do we have?"

→ Asystole / PEA: If PEA: Carotid or femoral pulse control, resume CPR immediately, administer Suprajet 1mg iv, repeat administration every 3-5min

→ Ventricular Fibrillation / Ventricular Tachycardi[redacted]*hock with 200J, resume CPR immediately, go to Checklist 1*

Go back to ❹ and maintain rhythm analysis – shock – CPR cycle

► mind Essential box

► blood gas analysis / blood sample

► evaluate possible Causes (Hs / Ts) ► perform FEEL (Instructions)

► if Asystole perform technical re-check

► transportation under CPR or prolonged CPR ► consider LUCAS or ECMO/ECLS

❺ ROSC ► see post-reanimation protocol

(Boxes are not listed again as they contain same content as in V.II)

7.4 Haemodynamic unstable Tachyarrhythmia with Pulse

3 Instabile Tachykardie mit Puls

UNIVERSITÄTS KLINIKUM FREIBURG

persistierende Tachykardie ▶ HF > 100/min + 1 weiteres: Schock, Synkope, Vigilanzänderung, anhaltende AP, akute Herzinsuffizienz ☎ Heilmeyer: 34915

START

❶ Laut: **„Wer ist Teamleiter?“**

❷ O_2-Gabe

❸ peripheren Zugang sichern

❹ **Kardioversion**

▶ Kurznarkose mit **Dipidolor + Dormicum** (**MEDIKAMENTE**)

▶ Narkoseerfolg prüfen

▶ Durchführung s. ANLEITUNG

▶ während Schock O_2-Gabe unterbrechen

▶ nach dem 3. Schock Cordarex 300mg i.v.

❺ Mögliche **URSACHEN** evaluieren

▶ BGA / Labor anfordern, Echo durchführen

❻ Nach erfolgreicher Kardioversion: Cordarex 900mg über 24h i.v.

MEDIKAMENTE

**Kurznarkose**	
Dormicum	bei 70kg: **2mg** i.v.-Bolus und bis Effekt titrieren
Dipidolor	bei 70kg: **15mg** i.v.-Bolus (= 1 Amp.) und bis Effekt titrieren
Amiodaron	Cordarex **300mg** i.v. über 10-20min<br>Erhaltungsdosis 900mg/24h i.v.
Magnesium	bei Torsade-de-pointes 2g i.v. über 10min

ANLEITUNG Kardioversion

1 Defi-Pads **(Abb. 1)** und seperate EKG-Elektroden aufkleben

2 Defi auf Defib-Modus einstellen

3 Synchronisation: **„SYNC“**-Taste betätigen **(Abb. 2)**

4 Energie-Niveau auf **120J** einstellen

5 Laden und Schock abgeben

6 Falls Tachykardie persistiert Energielevel steigern (120J - 150J - 200J)
Bei jedem Schock muss die „SYNC“-Taste erneut betätigt werden!
CAVE Falls Synchronisation nicht möglich: Unsynchronisierte Schocks mit 200J abgeben

Abb. 1

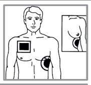

Abb. 2

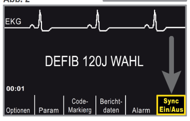

3

mögliche URSACHEN

- Myokardinfarkt
- Elektrolytstörung (s.u.)
- Herzinsuffizienz, Kardiomyopathie
- Lungenembolie
- Intoxikation
- Säure-Basen-Störung
- Myo - / Perikarditis (selten)
- Thyreotoxische Krise (selten)

Elektrolytstörung

Hyperkaliämie

- 20IE Inuslin in 200ml 20%-Glucose i.v. über 20min
- 10%-Ca-Gluconat 10ml i.v. über 3min, nach 5min wiederholen
- 10mg Salbutamol über Vernebler-Maske (nicht als Monotherapie)
- Bei pH < 7,2 Na-bicarbonat 50mmol über 5min (nicht als Monotherapie)

Hypokaliämie

K-Chlorid 40mval / l Jono i.v.

Persistent Tachycardia ► Heart rate > 100/min + 1 other: haemodynamic shock, altered vigilance, persistent angina pectoris, acute heart failure ☎ ICU

START

❶ *Loud: “Who is team leader?”*

❷ *Administer Oxygen*

❸ *Insert peripheral venous catheter*

❹ *Cardioversion:*

► *Short anaesthesia with Midazolam + Piritramide (Drugs)*

► *check success of anaesthesia*

► *see Instructions for execution*

► *stop oxygen flow when shock is administered*

► *after 3rd shock administer Cordarex 300mg iv*

❺ *evaluate possible Causes*

► *blood gas analysis, blood sample, ultrasound*

❻ *after successful cardioversion: Cordarex 900mg iv over 24 hours*

DRUGS & DOSING

Short anaesthesia

Midazolam	*for 70 kg: 2mg iv bolus injection and titrate to effect*
Piritramide	*for 70 kg: 15mg iv bolus injection and titrate to effect*
Amiodarone	*Cordarex® iv over 10-20min*<br>*Maintenance dose: 900mg/24h iv*
Magnesium	*in case of Torsade-de-pointes 2g iv over 10min*

INSTRUCTIONS: Cardioversion

1. *Attach defibrillator pads (fig. 1) and ECG electrodes*
2. *Turn defibrillator to Defib-Mode*
3. *Synchronisation: press **"SYNC"** button*
4. *Turn energy level to **120J***
5. *Charge and administer shock*
6. *If tachycardia persists increase energy level (120J – 150J – 200J)*

*"SYNC" button must be pressed prior to **EVERY** following shock*
***CAVE**: If no synchronisation possible: administer unsynchronised shocks with 200J*

Possible CAUSES

- *Myocardial infarction*
- *Electrolyte imbalance*
- *Heart failure, Cardiomyopathy*
- *Pulmonary embolism*
- *Intoxication*
- *Acid-base-imbalance*
- *Myo – / pericarditis (rare)*
- *Thyrotoxic crisis (rare)*

Electrolyte Imbalance

Hyperkalaemia

- *20 IE insulin in 200ml 20%-glucose, iv-administration over 20min*
- *10ml of 10% Calcium gluconate, iv-administration over 3min, repeat after 5min*
- *Additional (not suited as single therapy): 10mg salbutamol over nebuliser*
- *Additional if pH < 7,2 (not suited as single therapy): iv-administration of sodium bicarbonate 50mmol over 5min*

Hypokalaemia

- *40mval of potassium chloride in 1l balanced eletrolyte solutions, slow iv-administration*

7.5 Haemodynamic unstable Bradyarrhythmia with Pulse

4 Instabile Bradykardie mit Puls

UNIVERSITÄTS KLINIKUM FREIBURG

persistierende Bradykardie ▶ HF < 60/min + 1 weiteres: Hypotonie, Synkope, Vigilanzänderung, anhaltende AP, akute Herzinsuffizienz ☎ Heilmeyer: 34915

START

❶ Laut: **„Wer ist Teamleiter?“**

❷ O_2-Gabe

❸ peripheren Zugang sichern ▶ **Atropin 0,5mg i.v.-Bolus**

❹ **Transkutanes Pacing**

- ▶ Kurznarkose mit **Dipidolor + Dormicum** (**MEDIKAMENTE**)
- ▶ Narkoseerfolg prüfen
- ▶ Durchführung s. **ANLEITUNG**

❺ mögliche **URSACHEN** evaluieren

- ▶ BGA / Labor anfordern, Echo durchführen

MEDIKAMENTE

Atropin	0,5mg i.v.-Bolus, evtl. weitere i.v.-Boli mit 0,5mg bis Maximaldosis 3mg
**Kurznarkose**	
Dormicum	bei 70kg: **2mg** i.v.-Bolus und bis Effekt titrieren
Dipidolor	bei 70kg: **15mg** i.v.-Bolus (= 1 Amp.) und bis Effekt titrieren

ANLEITUNG Transkutanes Pacing

1. Defi-Pads **anterior-posterior (Abb. 1)** und separate EKG-Elektroden aufkleben
2. Defi auf **SCHRITTMACHER-Modus** umstellen
3. Frequenz ist auf **70/min** eingestellt **(Abb. 2)**
4. Reizimpulsmarkierung sichtbar **(Abb. 3)**?
5. mit **70mA** beginnen und zügig in 5mA-Schritten steigern **(Abb.2)**
6. Stimulation wirksam: verbreiterte QRS-Komplexe nach jeder Impulsmarkierung **(Abb.4)** und tastbarer Puls in A. femoralis
7. Schrittmacherstrom um 20mA ausgehend vom Capture-Strom steigern

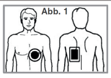
Abb. 1

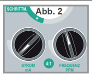
Abb. 2

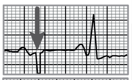
Abb. 3

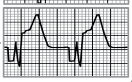
Abb. 4

4

mögliche URSACHEN

- Myokardinfarkt
- Elektrolytstörung (s.u.)
- Intoxikation (s.u.)
- Lungenembolie
- Herzinsuffizienz, Kardiomyopathie, Myokarditis
- Bradyarrythmie bei VHF
- SA- oder AV-Block
- Säure-Basen-Störung
- Hypoxie, gesteigerter Hirndruck
- Sick-Sinus-Syndrom (selten)

Elektrolytstörung

Hyperkaliämie
- 20IE Inuslin in 200ml 20%-Glucose i.v. über 20min
- 10%-Ca-Gluconat 10ml i.v. über 3min, nach 5min wiederholen
- 10mg Salbutamol über Vernebler-Maske (nicht als Monotherapie)
- Bei pH < 7,2 Na-bicarbonat 50mmol über 5min (nicht als Monotherapie)

Hypokaliämie
K-Chlorid 40mval / l Jono i.v.

Medikamenten-Intoxikation

Beta-Blocker	Glucagon 2-5mg i.v. über 1-2min, Erhaltungsdosis 2-5mg/kg/h
Digitalis	Fab-Antikörperfragmente (Dosis nach Digitalis-Konzentration)
Ca-KB	Ca-Gluconat 10% 30-60ml i.v. oder Ca-Chlorid 5,5% 15-30ml i.v.

Persistent Bradycardia ▶ Heart rate < 60/min + 1 other: haemodynamic shock, altered vigilance, persistent angina pectoris, acute heart failure ☎ ICU

START

❶ *Loud: “Who is team leader?”*

❷ *Administer Oxygen*

❸ *Insert peripheral venous catheter ▶ administer Atropine 0,5mg as iv bolus injection*

❹ *Transcutaneous Pacing:*

▶ *Short anaesthesia with Midazolam + Piritramide (Drugs)*

▶ *check success of anaesthesia*

▶ *see Instructions for execution*

❺ *evaluate possible Causes*

DRUGS & DOSING

Atropine *0,5mg iv bolus injection*
further bolus injections with 0,5mg possible up to maximum dose of 3mg

Short anaesthesia

Midazolam *for 70 kg: 2mg iv bolus injection and titrate to effect*
Piritramide *for 70 kg: 15mg iv bolus injection and titrate to effect*

INSTRUCTIONS: Transcutaneous Pacing

1. *Attach defibrillator pads **anterior-posterior** (fig. 1) and ECG electrodes*
2. *Turn defibrillator to Pacemaker-Mode*
3. *Set Rate to **70/min** (fig. 2)*
4. *Stimulus visible (fig. 3)?*
5. *Start with amperage **70mA** and quickly increase in 5mA steps (fig. 2)*
6. *Capture: broad QRS complexes after every stimulus (fig. 4)*
7. *When capture is reached increase amperage by 20mA*

Possible CAUSES

- *Myocardial infarction*
- *Electrolyte imbalance*
- *Intoxication*
- *Pulmonary embolism*
- *Heart failure, Cardiomyopathy*
- *Bradyarrhythmia in atrial fibrillation*
- *SA- or AV-block*
- *Acid-base imbalance*
- *Hypoxia, increased cerebral pressure*
- *Sick-Sinus-Syndrome (rare)*

Electrolyte Imbalance

Hyperkalaemia

- *20 IE insulin in 200ml 20%-glucose, iv-administration over 20min*
- *10ml of 10% Calcium gluconate, iv-administration over 3min, repeat after 5min*
- *Additional (not suited as single therapy): 10mg salbutamol over nebuliser*
- *Additional if pH < 7,2 (not suited as single therapy): iv-administration of sodium bicarbonate 50mmol over 5min*

Hypokalaemia

- *40mval of potassium chloride in 1l balanced eletrolyte solutions, slow iv-administration*

Drug Intoxication

Beta blockers	*Glucagon 2-5mg, iv-administration over 1-2min, maintenance dose: 2-5mg/kg/h*
Digitalis	*Fab antibody fragments*
Calcium channel blockers	*30-60ml of 10% Calcium gluconate iv or*<br>*15-30ml 5,5% Calcium chloride iv*
